# Effects of Replication and Transcription on DNA Structure-Related Genetic Instability

**DOI:** 10.3390/genes8010017

**Published:** 2017-01-05

**Authors:** Guliang Wang, Karen M. Vasquez

**Affiliations:** Division of Pharmacology and Toxicology, College of Pharmacy, The University of Texas at Austin, Dell Pediatric Research Institute, 1400 Barbara Jordan Blvd., Austin, TX 78723, USA; karen.vasquez@austin.utexas.edu

**Keywords:** non-B DNA, genetic instability, replication, transcription, collision, DNA repair

## Abstract

Many repetitive sequences in the human genome can adopt conformations that differ from the canonical B-DNA double helix (i.e., non-B DNA), and can impact important biological processes such as DNA replication, transcription, recombination, telomere maintenance, viral integration, transposome activation, DNA damage and repair. Thus, non-B DNA-forming sequences have been implicated in genetic instability and disease development. In this article, we discuss the interactions of non-B DNA with the replication and/or transcription machinery, particularly in disease states (e.g., tumors) that can lead to an abnormal cellular environment, and how such interactions may alter DNA replication and transcription, leading to potential conflicts at non-B DNA regions, and eventually result in genetic stability and human disease.

## 1. Introduction

Genomic DNA is the macromolecule responsible for the storage of genetic information; it is used for replicating another copy of genomic DNA for a daughter cell, and codes for other functional macromolecules such as messenger RNA for protein synthesis and regulatory non-coding RNAs in cells—i.e., DNA replication and transcription. Maintaining the accuracy and integrity of DNA is crucial for cell survival and, thus, cells have developed highly sophisticated DNA damage-monitoring and repair systems to avoid mutation to maintain genome integrity. Interestingly, the distribution of mutations in human genomes is not random, and many mutational “hotspots” that have been identified in disease etiology are often located in specific repetitive, non-B DNA-forming sequences [1,2,3,4,5,6,7,8,9,10].

## 2. Repetitive Sequences, Non-B DNA Conformations and Genetic Instability

### 2.1. B-DNA and non-B DNA Structures

After years of research from many groups, Watson and Crick described a well-accepted model of the DNA double-helical structure, referred to as B-DNA, where the two complementary strands align in an antiparallel, right-handed orientation, held together by hydrogen bonding between each of the base-pairs [11]. The double-stranded DNA likely exists in a B-form conformation in cells the majority of the time and is further organized into higher orders of structures, including wrapping of DNA around histone cores to form nucleosomes and then further wrapped and condensed into chromatin. All DNA metabolic processes in cells, including DNA replication, transcription, recombination, and repair occur within the background of DNA secondary structure and tertiary chromatin conformations.

**Figure 1:**
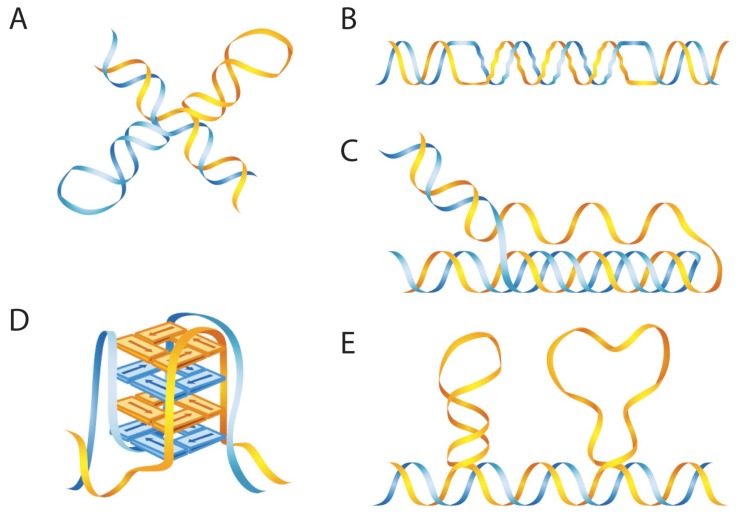
Schematic of several commonly studied non-B DNA structures. (**A**) Cruciform; (**B**) Left-handed Z-DNA; (**C**) Intramolecular triplex H-DNA; (**D**) G-quadruplex/tetraplex; (**E**) Stem-loop (**left**) or bubble (**right**) formed at slipped DNA, (from [24], with permission).

The Human Genome Project revealed that >50% of human genomic DNA consists of repetitive sequences, containing repeating units of different lengths ranging from single base-pairs to large segments of DNA at a mega base-pair (Mbp) scale [12]. These repetitive elements were originally considered as “by-products” of genome evolution or locations of viral attack and were often referred to as “junk DNA”. However, we have now realized that these repetitive elements play important regulatory roles in genomic structure and function. Notably, many of these repeats are able to form alternative secondary DNA conformations that differ from the classic B-DNA structure, due to inter- and intra-molecular interactions within/between the repetitive elements. Figure 1 depicts several commonly studied non-B DNA conformations. More than 15 types of “non-B” DNA structures that differ from B-DNA have been characterized to date [6,13,14]. For most types of non-B DNA structures, the first step of the conformational transition from B-form DNA to non-B is separation of the DNA duplex into single-stranded DNA (ssDNA), providing the single-stranded repetitive sequence the opportunity to interact with nucleotides that are on the same strand or with those of the underlying duplex regions. For example, a single-stranded inverted repeat sequence can form Watson-Crick base-pairs between the self-complementary regions on the same strand to form hairpin or cruciform structures [15]; an H-DNA structure can form at a purine- or pyrimidine-rich ssDNA region with mirror repeat symmetry that can fold back and Hoogsteen hydrogen bond to the major groove of the other half of the duplex containing mirror symmetry [16,17]. If the ssDNA contains four guanine runs, each containing three or more guanines, the ssDNA can fold into a G-tetrad structure via Hoogsteen-hydrogen bonding to form a square planar structure [18], and three or more of such stacked guanine tetrads are referred to as G-quadruplex or G4 DNA structures [19,20]. ssDNA containing simple repetitive units can re-anneal with the complementary strand with misalignment, resulting in loop structures [21]. Z-DNA is a left-handed helix that can form in regions of alternating purine-pyrimidine sequences, where the guanines in every two base-pairs are in the *syn* conformation, in contrast to the canonical anti-conformation in B-DNA, which twists the phosphodiester backbone into a zigzag (hence the name) pattern [22,23]. A-DNA is a duplex structure that exits under dehydrating conditions such as crystal formation, with altered major and minor groove structures [6].

Formation of non-B DNA structures has been characterized using many different techniques such as circular dichroism (CD) that provide signature spectropolarimetry for a non-B DNA structure under specific conditions of temperature, ionic strength, and pH [25]; enzyme or chemical probing, or the use of structure-specific antibodies that can potentially recognize and probe for specific DNA conformations [26,27,28,29]; and direct visualization of some non-B DNA structures that induce DNA strand bending under electron microscopy [30,31,32,33,34]. These techniques have provided strong evidence for the presence of non-B DNA both in vitro and in vivo.

### 2.2. Non-B DNA-Forming Sequences Can Lead to Genetic Instability

Non-B DNA-forming sequences are involved in many important biological functions such as DNA replication, gene regulation, recombination, epigenetic modification and chromatin structure formation (for reviews, see [6,14,35,36,37]). It is now well accepted that non-B DNA-forming sequences, in the absence of exogenous DNA damaging factors, can lead to genetic instability in both prokaryotic and eukaryotic cells [14,38,39,40,41,42]. Expansion of tri-nucleotide repeats such as CAG/CTG, CGG/CCG, GAA/TTC is responsible for more than 30 human neurological diseases [40,41,43,44], and sequences that can form H-DNA, Z-DNA, hairpin, cruciform, and G4-DNA structures are enriched at, and often colocalize with, mutation hotspots in cancer and other diseases [45,46,47,48,49,50,51,52,53,54,55,56,57,58,59,60]. We discovered that short repetitive sequences that can form H-DNA, Z-DNA or cruciform structures are intrinsically mutagenic in bacteria, yeast, mammalian cells and in mouse genomes [4,14,61,62,63]. We have summarized in review articles how non-B DNA conformations may be recognized as “DNA damage” and processed via DNA repair proteins in replication-dependent or replication-independent mechanisms [38,64]. However, both DNA replication and transcription are critical factors involved in DNA structure formation and non-B DNA-induced genetic instability and will be discussed in detail below.

## 3. DNA Replication and Transcription Facilitate Non-B DNA Structure Formation

In the classical B-formation, DNA duplexes are perfectly base-paired, and thus are in the most stable energy status; the chromatin organization also favors maintaining DNA in the B-formation. Negative supercoiling can in general facilitate the B-DNA to non-B DNA transition because it can provide energy to destabilize the B-DNA structures, stimulate DNA breathing, a spontaneous duplex separation below the DNA melting temperature, and exposure of ssDNA. As mentioned above, opening of the B-DNA duplex and exposing ssDNA is typically required for the transition from B-DNA to non-B DNA conformations. Therefore, any DNA metabolic event that involves the generation of negative supercoiling, separation of the duplex, and/or exposure of ssDNA in appropriate regions may facilitate the formation of non-B DNA structures.

### 3.1. DNA Replication and non-B DNA Formation

DNA replication requires opening of chromatin structures and unwinding of the packed DNA from nucleosome cores, employing DNA helicases to separate the DNA duplex into two ssDNA regions, and topoisomerases to relax the positive supercoiling generated upstream. The migration of replication forks through non-B DNA-forming sequences can generate negative supercoiling downstream that can facilitate the formation of non-B DNA structures. For example, triplet repeats can form hairpin or loop structures on ssDNA via intrastrand hydrogen bonding, which can result in repeat unit expansion and human disease [65]. It has been found that the expansion of these repeats was much more pronounced in highly proliferative tissues [66,67] or in rapidly dividing cells [68,69]. Thus, DNA replication appears to play important roles in the generation of non-B DNA conformation and the subsequent genetic instability.

DNA replication generates long ssDNA regions during lagging strand synthesis and creates a favorable environment for the formation of non-B DNA structures during replication [70]. Single-stranded DNA binding protein (SSB) in prokaryotic cells or replication protein A (RPA) in eukaryotic cells binds to the ssDNA and can prevent non-B DNA structure formation [71,72,73]. However, the highly dynamic feature of RPA or SSB binding to ssDNA can grant the formation of high-order DNA confirmations at appropriate regions, particularly with the assistance of negative supercoiling and/or non-B DNA-associated proteins [74,75,76,77]. Therefore, the location and orientation of the triplet repeat relative to the replication origin can have dramatic effects on the stability and the types of mutations induced at these sequences [78,79,80,81,82,83,84,85,86,87]. CTG repeats form a slightly more stable hairpin structure than the CAG repeats on the complementary strand [88]. Correspondingly, large expansions of CTG/CAG triplet repeats occur predominantly when the CTG repeats serve as the template for the leading strand replication; and deletions are more prominent when the CTG repeats are on the template for lagging strand synthesis [78]. In a study using plasmids containing an SV40 replication origin, Cleary et al. found that CAG(79) repeats yielded predominantly expansions when the repeats were positioned at the 3′ end of an Okazaki initiation zone, and mostly contractions when the repeats were located at the 5′ end [89]. In yeast, a long track of 78–150 trinucleotide GAA repeats in the intron of the *URA3* gene caused substantial repeat expansion, and the number of repeat units added in these long GAA tracts were in a relatively narrow range of 44–63 triplets, which is approximately the length of an Okazaki fragment in yeast [90]. The threshold of repeat length approximately matches the length of Okazaki fragments in various species. In bacterial cells, the Okazaki fragment is longer, approximately 1000 bp. It was found that the majority of CTG repeat expansions were incremental when the CTG tracks were shorter than an Okazaki fragment (120–200 repeats); and when CTG repeats were longer than 1000 bp (330–500 repeats), the majority of expansions were discrete changes in the repeat size, adding hundreds or thousands of base-pairs but nothing in between [91]. Mutation of polymerase α, which can slow DNA replication and lead to longer stretches of ssDNA in the Okazaki initiation zone, dramatically increased the expansion events and the size of repeat expansions, accompanied by mutations outside of the repetitive sequence that inactivated the *URA3* gene [90]. In fact, mutations in other genes involved in DNA replication, e.g., *Flap endonuclease 1* (*FEN-1*) [92] or DNA polymerases [93], have significant effects on triplet repeat stability. These data provide some examples of the effects of DNA replication on non-B DNA formation and non-B-induced genetic instability.

### 3.2. Transcription and non-B DNA formation

The transcription machinery also unwinds DNA from nucleosomes, separates the coding and non-coding strands and generates negative supercoiling downstream [94]. These conditions can favor non-B DNA conformations during transcription. For example, Wittig et al. found that transcription through the human *c-MYC* gene was required for Z-DNA formation in the promoter regions, as evidenced by the binding of Z-DNA-specific antibodies to this region only when the gene was actively transcribed [54,95,96]. In isolated nuclei of *Allium cepa* L. root meristems, labels for Z-DNA structures nearly disappeared when RNA polymerase (I and II)-dependent transcription was inhibited, supporting the idea that transcription was required for Z-DNA formation. In fact, the authors suggested using the in situ immunodetection of Z-DNA as a marker of transcription [97].

During transcription, nascent single-stranded RNA can bind back to the template DNA strand to form an R-loop conformation, which contains an RNA-DNA hybrid and a long region of ssDNA. If the ssDNA region is comprised of a repetitive element and can fold into a higher-order structure such as G4-DNA or a hairpin structure, the “R-loop + non-B DNA complex” could be further stabilized [34,98]. There are excellent review articles in this issue on this topic, and thus will not be further discussed here.

## 4. Non-B DNA Conformations Impact DNA Replication and Transcription

Both DNA replication and transcription require DNA to be in a ssDNA status to serve as a template so each incoming deoxynucleoside triphosphate (dNTP) (for DNA replication) or ribonucleoside triphosphate (for transcription) can form an appropriate base-pair with a base in the template strand before polymerization to the 3′ end of a polynucleotide chain to form a complementary DNA or RNA strand. Therefore, it is reasonable to speculate that any impediments to the access of the ssDNA template could affect the progression and/or the fidelity of the DNA and RNA polymerases.

### 4.1. Non-B DNA Structures in front of Replication and Transcription machineries

Non-B DNA structures formed in front of replication or transcription machineries have been shown to affect both the processivity and fidelity of these events. In cells, when a DNA or RNA polymerase encounters a non-B DNA structure, to progress or to stall is the result of competition between the stability of the non-B DNA conformation and the ability of components in the polymerase complexes to remove or pass through the impediment caused by the non-B conformation. An intermolecular triplex (similar in structure to an intramolecular H-DNA triplex) structure formed between two oligonucleotides has been shown to inhibit DNA unwinding in vitro by the eukaryotic SV40 large T-antigen DNA helicase [99], and such inhibitory effects were diminished when the pH was increased to 8.7, which did not favor the formation of the triplex structure [99]. Although the same group reported that a third-strand TC(20) oligonucleotide in a triplex structure could be separated by the SV40 large T-antigen DNA helicase from a linearized double-stranded plasmid DNA substrate containing a GA(27) repeat with the energy provided by ATP hydrolysis, it happened only when 3′-flanking ssDNA was available on the third-strand oligonucleotide. Because 3′-flanking ssDNA was required for large T-antigen helicase to load and migrate in a 3′--> 5′ direction toward the triplex region before it released the third strand [100], it is likely that such helicases are not able to unwind intramolecular H-DNA where no 3′-free end is available.

**Figure 2:**
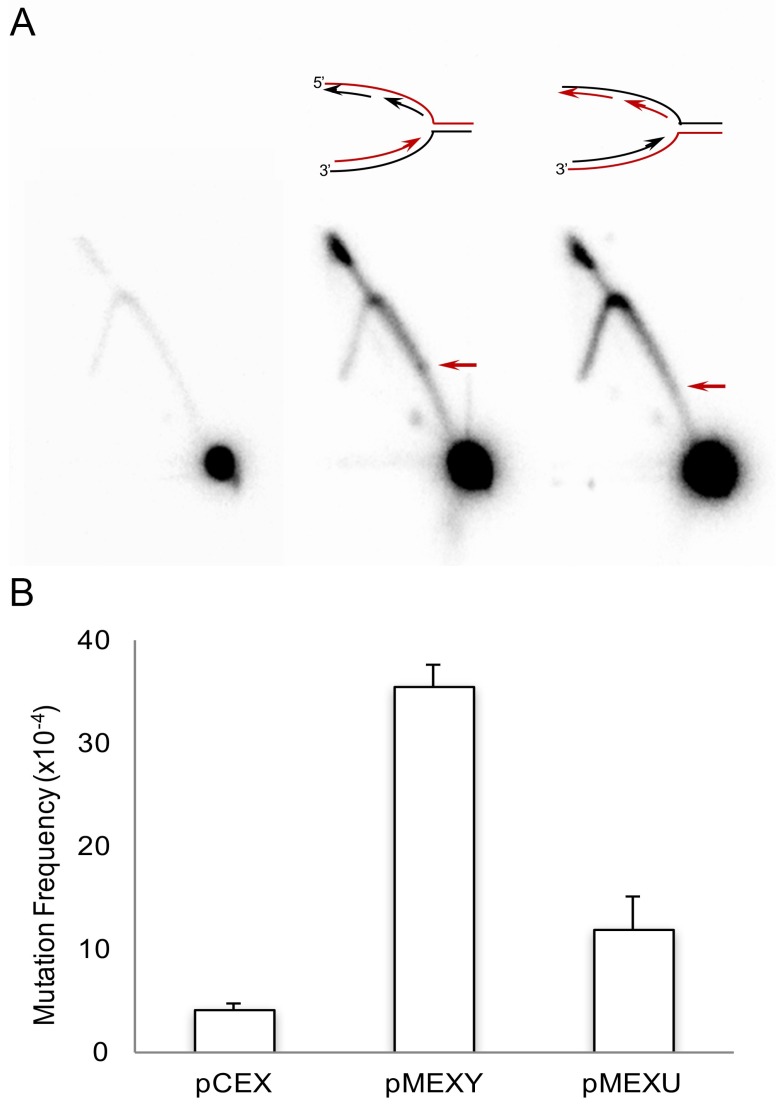
Effects of H-DNA orientation on DNA replication and genetic instability in mammalian cells. Reporter shutter vectors, pCEX and pMEXY and pMEXU, were transfected into mammalian COS-7 cells. The SV40 replication origin on the plasmids supports bi-directional replication in COS-7 cells. (**A**) Effects of orientation on H-DNA-induced replication stalling in mammalian COS-7 cells. Replication intermediates of plasmids were recovered 24 h post-transfection and were separated via 2-D gel electrophoresis. The fragments containing the H-DNA-forming or control sequences were probed by Southern blotting. The arrow designates the bulge on the Y-shaped replication arc, indicative of the accumulation of stalled replication intermediates. A representative image of three independent repeats is shown; (**B**) Effects of orientation on H-DNA-induced mutation frequencies in mammalian COS-7 cells. Mutation-reporter plasmids were recovered 48 h post-transfection, followed by DpnI digestion to remove the un-replicated plasmid DNA. Mutants generated were screened in indicator bacterial MBM7070 cells. Error bars show the standard errors of the mean value of three independent experiments.

There are many reports demonstrating that non-B DNA can cause replication fork stalling [80,101,102,103,104,105,106,107], which can result in replication fork collapse, and the formation of DNA double-strand breaks (DSBs), and subsequent genetic instability [108,109,110]. We have discovered that a short 34 bp H-DNA-forming sequence from the human *c-MYC* promoter, near one of the translocation hotspots found in Burkitt lymphoma, was mutagenic on reporter plasmids in human cells and on chromosomes in mouse genomes via both replication-dependent and replication-independent mechanisms [38,61,62]. We found that this repetitive element stalled DNA replication fork progression in mammalian cells, and also stimulated the formation of DSBs and mutations in the presence or absence of DNA replication [24]. Using the same approach, we report here that the orientation of this polypurine-polypyrimidine mirror repeat plays an important role in replication stalling and mutation in mammalian cells in a replication orientation-dependent fashion (Figure 2). The same repeat was inserted at the same location on the mutation-reporter plasmid, but in opposite directions, i.e., the purine-rich strand served as the template for lagging strand synthesis in pMEXY, and the pyrimidine-rich strand was used for lagging strand synthesis in pMEXU. The plasmids (pMEXY, pMEXU and control B-DNA-forming pCEX) were transfected into mammalian COS-7 cells, and replication intermediates were recovered 24 h later and subjected to 2-D electrophoresis of DNA replication intermediates as previously described [4,24,111]. The results revealed a typical Y-shape replication arc because the SV40 origin was not included in the probed area. As shown in Figure 2A, DNA replication was stalled at the H-DNA region in the pMEXY plasmid and resulted in bulges on the right arm of the arc, and a much lighter left arm, suggesting fewer replication intermediates past the H-DNA sequence compared to that in pMEXU. Notably, the replication stalling on the pMEXU plasmid was not as obvious as that on pMEXY, suggesting a stronger impact on DNA replication when the purine-rich strand was on the lagging strand. Consistently, H-DNA-forming sequences induced higher mutation frequencies than control B-DNA sequences in the same reporter plasmids 48 h after transfection into mammalian COS-7 cells, and pMEXY stimulated higher mutation frequencies than pMEXU (Figure 2B). This result provided evidence for a role of non-B DNA-induced replication fork stalling in DNA-structure-induced genetic instability in mammalian cells.

Stalled transcription complexes can also cause genetic instability. Transcription-coupled DNA repair (TCR) is a pathway that preferentially repairs DNA lesions in the template strand over the non-template strand and results in the excision of a fragment of the DNA containing the lesion. In TCR, RNA polymerase stalling, rather than DNA damage per se, can serve as a signal for triggering DNA repair [112]. Thus, we and others have proposed that a stalled RNA polymerase at non-B DNA structures, even in the absence of DNA damage per se, may be sufficient to trigger a “gratuitous” DNA cleavage and repair. A “successful repair and re-synthesis” would rebuild the repetitive sequence and the non-B DNA structure could reappear when transcription or replication occurred, leading to another round of “gratuitous” DNA cleavage and repair until an error is generated during this process, resulting in DNA breakage and mutation.

### 4.2. Non-B DNA Structures behind Transcription Machinery

Processivity and fidelity of RNA polymerases may be affected by non-B DNA structures formed behind the transcription machinery [113,114,115]. In a collaborative project with the Hanawalt group, we found that transcription by T7 RNA polymerase was paused at an H-DNA-forming sequence in a fraction of molecules within and even downstream of the H-DNA-forming sequence from the human *c-MYC* promoter (same as the H-DNA-forming sequence used in Figure 2). We also found similar results with Z-DNA-forming CpG repeats, and the stalling was much more obvious in multiple round transcription [113,114]. Similarly, G-rich sequences that can form triplexes, G-quadruplexes, or R-loops can significantly block transcription by T7 RNA polymerase or mammalian RNA polymerase II. The replication stalling at G-rich sequences was orientation-, length- and supercoiling-dependent, implicating non-B DNA structures, rather than linear sequences per se, in transcription stalling [116]. Although stalling sites were observed both in front of and after G-rich sequences, the major stalling events occurred when RNA polymerase had passed G-rich inserts, supporting a model of R-loop structure formation where nascent RNA interacts with repetitive DNA sequences to form an RNA-DNA hybrid. Further, the non-B DNA structures formed at the G-rich DNA regions could further stabilize the interactions and impact the movement of RNA polymerase complexes [116]. Such effects may not only interfere with the functions of RNA polymerases, but also cause genetic instability as discussed above.

## 5. Non-B DNA Structures Cause Transcription and Replication Collision and Lead to Genetic Instability

In both prokaryotic and eukaryotic genomes, DNA replication and transcription could occur on the same DNA strand simultaneously, so it is possible that the two complexes could collide with each other in a “co-direction” fashion or “head-on”. Mirkin et al. reported that when collision or conflict occurred, replication fork progression was dramatically stalled at the transcribed DNA segments, suggesting a plausible direct contact between the two machineries [117]. DNA topological distortion (e.g., positive supercoiling) generated in front of both complexes might also serve as a barrier for further progression [118]. In addition, the active transcription machinery might recruit and increase the deoxyuridine triphosphate (dUTP) concentration near the DNA replication machinery and result in mis-incorporation of dUTP at the sites of deoxythymidine triphosphate (dTTP). The removal of dUTP would leave apurinic/apyrimidinic sites near the area where replication and transcription meet [119], such that collision could be detrimental to cells, resulting in genomic instability.

**Figure 3:**
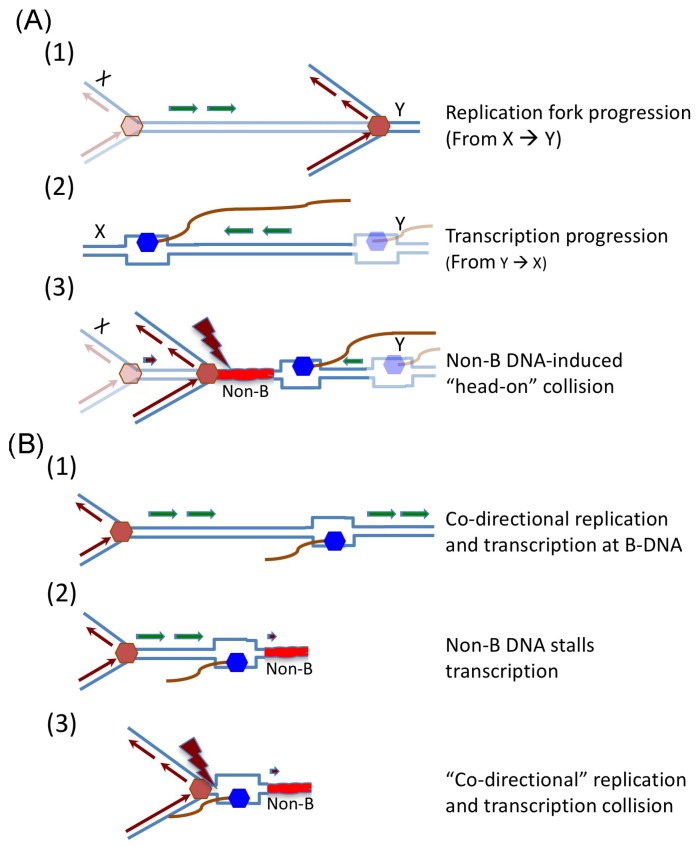
Models of non-B DNA-induced replication and transcription collision at repetitive regions. (**A**) Schematic diagram of non-B DNA-induced “head-on” replication-transcription collision. (1) DNA replication progresses from chromosome location “X” to “Y”; (2) Transcription progresses within the same region, but from the opposite direction (from “Y” to “X”) after (or before) DNA replication is complete in that area; (3) A non-B DNA structure formed between chromosome loci “X” and “Y” interrupts the initiation and/or progression of replication and transcription, resulting in “head-on” replication-transcription collision (shown as a lightning bolt). (**B**) Schematic diagram of non-B DNA-induced “co-directional” replication-transcription collision. (1) DNA replication and transcription occur simultaneously and “co-directionally” on the chromosome without collision; (2) A non-B DNA structure formed in front of the transcription machinery stalls transcription progression; (3) A DNA replication fork runs into the transcription machinery, resulting in a “co-directional” replication-transcription collision (shown as a lightning bolt).

In both prokaryotic and eukaryotic cells, transcription and replication are carefully organized, regulated, and timed. In prokaryotic genomes DNA replication is initiated at a single origin, and the highly expressed genes and essential genes are located on the template for leading strand synthesis, thus replication and transcription progress in the same direction. Thus, “head-on” collisions at these important genes are largely avoided. Transcription itself does not appear to affect DNA replication elongation when the two processes occur in a co-directional orientation or do not exist in close proximity [117]. In eukaryotic genomes, both transcription and replication start from multiple sites and replication forks move in both directions in the genome, so DNA and RNA polymerases have the risk of competing for the same DNA template if not properly regulated. Because non-B DNA conformations may affect the initiation and timing of both replication and transcription, and stall the elongation process, it is possible that non-B DNA-forming elements could cause “traffic congestion” on the DNA template and increase the chance of collision, leading to genetic instability. Inappropriate initiation or elongation of either replication or transcription could lead to head-on or co-directional collisions, as illustrated in Figure 3.

Sequences that can form non-B DNA structures are enriched in both rare and common fragile sites in human genomes [120]. Rare fragile sites are characterized by an expansion of CGG repeats (which can form Z-DNA, quadruplex structures, hairpin/cruciform structures or loop-outs) or AT-rich minisatellite repeats (which can form hairpin or cruciform structures). Common fragile sites that are present in all individuals also contain many elements that can form non-B DNA, particularly AT-rich inverted repeats that can form hairpin or cruciform structures [120,121,122]. Fragile sites affect DNA replication and lead to chromosome breakage, and are associated with disease development [123,124,125]. One of the key features of fragile sites is that they are replicated slower and in later stages (there are also early replicating fragile sites, (ERFSs), see below), and non-B DNA conformations formed in these repetitive regions are considered as one of the contributors for abnormal DNA replication within these regions [120].

Notably, most of the common fragile sites lie within, or span, known genes and are transcribed in many cells [126]. Moreover, in a study performed by Calin et al. [127], more than 50% of the analyzed microRNAs were mapped near known fragile sites, with a nine-fold greater occurrence than in non-fragile control regions. Because many of these elements within the fragile sites serve as templates for both DNA replication and transcription, and can disrupt the timing and progression of both activities, the two machineries could meet at these repetitive sequences. It is of particular interest that many actively transcribed large human genes (spanning more than 1.0 Mb) contain chromosomal fragile sites [128]. Some large genes such as *fragile histidine triad* (*FHIT*, 1.5 Mb), *WW domain containing oxidoreductase* (*WWOX*, 1.1 Mb), and *IMP2 inner mitochondrial membrane peptidase-like* (*IMMP2L*, 0.9 Mb) contain repetitive sequences in common fragile sites, FRA3B, FRA16D and FRA7K, respectively. Helmrich et al. [129] found that transcription in these large genes was very slow (~30 nucleotides/second) and took more than 11–13 h to finish. Because transcription in these regions takes longer than a cell cycle (~10 h for the cells studied), collisions between replication and transcription machineries were very likely. RNA synthesis stalling within the fragile sites colocalized with genomic breakage hotspots. Under mild replication stress by aphidicolin, *FHIT* and *WWOX* induced chromosomal breakage in human B-lymphoblasts where the genes were expressed, but not in myoblasts where the genes were silent. The *IMMP2L* gene was expressed in both cell types, but higher in B-lymphoblasts, and the chromosomal lesions at the FRA7K fragile site were detected in both cell types and was ~3-fold higher in in B-lymphoblasts [129]. Interestingly, although RNA-DNA hybrids were detected at sites of replication and transcription overlap, RNase H2 did not affect fragility of these repetitive sequences when transcribed [129]. However, another study reported that in epithelial and erythroid cells, transcription did not play a major role in chromosomal breakage in fragile sites within large genes [130]. Whether or not replication and transcription were stalled and/or resulted in collisions within these fragile sites is not clear.

In B lymphocytes, Barlow et al. identified a recurrent early replicating chromosomal fragility termed early replication fragile sites (ERFSs) that cause replication fork stalling under hydroxyurea (HU)-induced stress [131]. G + C nucleotides and repetitive elements such as long interspersed elements (LINEs) and short interspersed elements (SINEs) are significantly enriched in ERFSs. These fragile sites are located in highly transcribed genes, and transcription can significantly increase the fragility of the ERFSs [131]. Ataxia telangiectasia and Rad3-related (ATR) kinase plays an important role in coordinating transcription and replication in eukaryotic cells by suppressing transcription and yielding the right of way to replication forks [132]. Inhibiting ATR activity in B lymphocytes also significantly increased the fragility seen at ERFSs [131], similar to what had been seen on common fragile sites. Therefore, these ERFSs may also lead to collisions by impacting both DNA replication and transcription.

In addition to transcription disruption and replication fork collapse, slowing of DNA replication by collision with transcription complexes could give rise to the formation of non-B DNA structures in adjacent areas if the collision occurs within repetitive sequence or within a continuous track of several repetitive elements. It was recently reported that in an artificial system to allow study of replication-transcription collisions in actively dividing bacteria, duplications/deletions and base substitutions were the two major classes of mutations that occurred at replication-transcription collision regions [133]. The duplications and deletions were significantly affected by transcription, and were likely caused by replication stalling events at collision sites where the replication fork first encountered a transcription complex [133]. These data suggested that when a collision occurred, the repetitive sequences in the templates for DNA replication (particularly for lagging strand synthesis), for transcription, and/or the nascent RNA strand containing the repeats may have had a greater chance to form non-B DNA conformations and RNA secondary structures. Such structure-on-structure complexes might introduce more complexity in resolving the collision and restarting the replication forks, resulting in more genetic instability events including repeat unit duplication or deletion, DNA breakage, and/or recombination.

## 6. Non-B DNA and Replication-Transcription Collision in Cancer

Cancer cells often exhibit a unique phenomenon of “replication stress”, where DNA replication fork progression in S phase is slower and the accuracy is reduced relative to normal cells. Although the mechanisms are not completely understood, replication stress in cancer cells may be the result of activation of oncogenes such as *resistance to audiogenic seizures (RAS)*, *v-myc avian myelocytomatosis viral oncogene homolog (MYC)*, *cyclin-dependent kinases (CDKs)* and *CYCLINs* [134,135,136,137,138,139,140,141,142,143,144]. These oncogenes can deregulate E2F-dependent G1/S transcription to stimulate S-phase entry before cells are ready for genome duplication [145,146]. The conditions generated by these oncogenes can also maintain E2F activity after S phase entry, which otherwise should be inactivated via a negative feedback loop [147], and manipulate DNA replication stress tolerance and genomic integrity [148]. Such inappropriately timed and slowed replication may overlap with transcription at regions containing repetitive sequences that form non-B DNA structures and lead to transcription-replication collision in actively transcribed regions as discussed above.

In fact, increased transcription and transcription-induced non-B DNA structure formation caused by activation of oncogenes may directly contribute to replication stress and chromosome instability in cancer cells [149]. The hormone estradiol (E2) can stimulate transcription of many E2-responsive genes and generate R-loops in transcribed regions. For example, Stork et al. found a significant enrichment and colocalization of R-loop formation, DSBs and gene rearrangement events (duplications, large deletions, inversions, and translocations) in breast cancer cells upon E2 treatment [150]. In human BJ fibroblast cells, overexpression of the *HRAS*^v12^ oncogene increased transcription-stimulated R-loop formation in many genes as evidenced by RNA-DNA hybrids, and caused replication stress. Transient suppression of transcription in *HRAS*^v12^ overexpressing cells using small molecule inhibitors or treating the cells with RNaseH1 to remove RNA/DNA hybrids overcame this replication stress [149]. The authors suggested that the formation of a “transcription-stimulated structural barrel” can significantly impact both transcription and replication progression. Thus, there is a possibility for collision and/or conflicts between the two machineries, which could result in subsequent chromosome instability.

Cancer cells are also known to have deregulated replication origin activities, including licensed origin scarcity during S phase due to lack of appropriate S-phase checkpoints and/or depletion of replicative DNA helicase minichromosome maintenance complex 2-7 (MCM2-7), and unscheduled replication caused by overexpression of proteins such as chromatin licensing and DNA replication factor 1 (CDT1) and cell division cycle 6 (CDC6). Licensed origin scarcity could result in insufficient DNA replication initiation, thus delayed or incomplete replication, and unscheduled replication could cause re-replication (i.e., segments of the genome replicated more than once) or premature DNA replication origin firing [144,145,146,151]. In both cases, deregulated replication could lead to a conflict or collision with the transcription machinery, resulting in genetic instability or cell death.

Telomestatin, a G-quadruplex stabilizer, is able to interrupt the loading of telomere maintenance proteins such as telomere-capping protein TRF2 and topoisomerase III alpha, and cause DNA damage, telomere instability and cell death [152,153,154,155]. Hasegawa et al. found that telomestatin-induced 53BP1 foci at telomeric regions and cell death depended on both DNA replication and transcription [156]. In fact, triggering replication stress in cancer cells as a potential cancer chemotherapeutic approach has been discussed previously [151,157,158,159,160]. Therefore, stimulating non-B DNA formation at actively transcribed regions by the use of small compounds [156,161] may represent a therapeutic strategy to enhance the efficiency and specificity of traditional chemotherapies.

## 7. Conclusions

In summary, studies on the impact of non-B DNA conformations on replication and transcription accuracy, efficiency, coordination and potential collisions have provided important information on mechanisms of genetic instability, although research in this field is still in its early stages. For example, a fine mapping of replication-transcription collision sites under different conditions, particularly in cancer cells under replication stress, will be very informative for exploring the contribution of non-B DNA structures in replication-transcription-related genetic instability and cell viability. Further work in this area is warranted to better understand the mechanisms involved and to develop potential approaches to reduce non-B DNA-induced replication-transcription conflicts in healthy cells or to stimulate such collision in cancer cells.

## References

[1] Chen X., Shen Y., Zhang F., Chiang C., Pillalamarri V., Blumenthal I., Talkowski M., Wu B.L., Gusella J.F. (2013). Molecular analysis of a deletion hotspot in the NRXN1 region reveals the involvement of short inverted repeats in deletion CNVs. Am. J. Hum. Genet..

[2] Smith D.G., Adair G.M. (1996). Characterization of an apparent hotspot for spontaneous mutation in exon 5 of the Chinese hamster APRT gene. Mutat. Res..

[3] De Graaff E., Rouillard P., Willems P.J., Smits A.P., Rousseau F., Oostra B.A. (1995). Hotspot for deletions in the CGG repeat region of FMR1 in fragile X patients. Hum. Mol. Genet..

[4] Lu S., Wang G., Bacolla A., Zhao J., Spitser S., Vasquez K.M. (2015). Short Inverted Repeats Are Hotspots for Genetic Instability: Relevance to Cancer Genomes. Cell Rep..

[5] Hsiao M.C., Piotrowski A., Alexander J., Callens T., Fu C., Mikhail F.M., Claes K.B., Messiaen L. (2014). Palindrome-mediated and replication-dependent pathogenic structural rearrangements within the NF1 gene. Hum. Mutat..

[6] Choi J., Majima T. (2011). Conformational changes of non-B DNA. Chem. Soc. Rev..

[7] Raynard S.J., Baker M.D. (2004). Cis-acting regulatory sequences promote high-frequency gene conversion between repeated sequences in mammalian cells. Nucleic Acids Res..

[8] Svetlova E.Y., Razin S.V., Debatisse M. (2001). Mammalian recombination hot spot in a DNA loop anchorage region: A model for the study of common fragile sites. J. Cell. Biochem..

[9] Waldman A.S., Tran H., Goldsmith E.C., Resnick M.A. (1999). Long inverted repeats are an at-risk motif for recombination in mammalian cells. Genetics.

[10] Hyrien O., Debatisse M., Buttin G., de Saint Vincent B.R. (1987). A hotspot for novel amplification joints in a mosaic of Alu-like repeats and palindromic A + T-rich DNA. EMBO J..

[11] Watson J.D., Crick F.H. (1953). The structure of DNA. Cold Spring Harb. Symp. Quant. Biol..

[12] Lander E.S., Linton L.M., Birren B., Nusbaum C., Zody M.C., Baldwin J., Devon K., Dewar K., Doyle M., FitzHugh W. (2001). Initial sequencing and analysis of the human genome. Nature.

[13] Wells R.D. (1988). Unusual DNA structures. J. Biol. Chem..

[14] Wang G., Vasquez K.M. (2006). Non-B DNA structure-induced genetic instability. Mutat. Res..

[15] Sinden R.R., Potaman V.N., Oussatcheva E.A., Pearson C.E., Lyubchenko Y.L., Shlyakhtenko L.S. (2002). Triplet repeat DNA structures and human genetic disease: Dynamic mutations from dynamic DNA. J. Biosci..

[16] Mirkin S.M., Frank-Kamenetskii M.D. (1994). H-DNA and related structures. Annu. Rev. Biophys. Biomol. Struct..

[17] Htun H., Dahlberg J.E. (1989). Topology and formation of triple-stranded H-DNA. Science.

[18] Sen D., Gilbert W. (1990). A sodium-potassium switch in the formation of four-stranded G4-DNA. Nature.

[19] Bochman M.L., Paeschke K., Zakian V.A. (2012). DNA secondary structures: Stability and function of G-quadruplex structures. Nat. Rev. Genet..

[20] Lane A.N., Chaires J.B., Gray R.D., Trent J.O. (2008). Stability and kinetics of G-quadruplex structures. Nucleic Acids Res..

[21] Djian P. (1998). Evolution of simple repeats in DNA and their relation to human disease. Cell.

[22] Malfoy B., Rousseau N., Vogt N., Viegas-Pequignot E., Dutrillaux B., Leng M. (1986). Nucleotide sequence of an heterochromatic segment recognized by the antibodies to Z-DNA in fixed metaphase chromosomes. Nucleic Acids Res..

[23] Johnston B.H. (1992). Generation and detection of Z-DNA. Methods Enzymol..

[24] Wang G., Zhao J., Vasquez K.M. (2016). Detection of cis- and trans-acting Factors in DNA Structure-Induced Genetic Instability Using In silico and Cellular Approaches. Front. Genet..

[25] Baase W.A., Johnson W.C. (1979). Circular dichroism and DNA secondary structure. Nucleic Acids Res..

[26] Agazie Y.M., Lee J.S., Burkholder G.D. (1994). Characterization of a new monoclonal antibody to triplex DNA and immunofluorescent staining of mammalian chromosomes. J. Biol. Chem..

[27] Raghavan S.C., Chastain P., Lee J.S., Hegde B.G., Houston S., Langen R., Hsieh C.L., Haworth I.S., Lieber M.R. (2005). Evidence for a triplex DNA conformation at the bcl-2 major breakpoint region of the t(14;18) translocation. J. Biol. Chem..

[28] Lee J.S., Burkholder G.D., Latimer L.J., Haug B.L., Braun R.P. (1987). A monoclonal antibody to triplex DNA binds to eucaryotic chromosomes. Nucleic Acids Res..

[29] Lee J.S., Latimer L.J., Haug B.L., Pulleyblank D.E., Skinner D.M., Burkholder G.D. (1989). Triplex DNA in plasmids and chromosomes. Gene.

[30] Mikheikin A.L., Lushnikov A.Y., Lyubchenko Y.L. (2006). Effect of DNA supercoiling on the geometry of holliday junctions. Biochemistry (Mosc.).

[31] Shlyakhtenko L.S., Potaman V.N., Sinden R.R., Lyubchenko Y.L. (1998). Structure and dynamics of supercoil-stabilized DNA cruciforms. J. Mol. Biol..

[32] Kurahashi H., Inagaki H., Yamada K., Ohye T., Taniguchi M., Emanuel B.S., Toda T. (2004). Cruciform DNA structure underlies the etiology for palindrome-mediated human chromosomal translocations. J. Biol. Chem..

[33] Vetcher A.A., Napierala M., Iyer R.R., Chastain P.D., Griffith J.D., Wells R.D. (2002). Sticky DNA, a long GAA.GAA.TTC triplex that is formed intramolecularly, in the sequence of intron 1 of the frataxin gene. J. Biol. Chem..

[34] Duquette M.L., Handa P., Vincent J.A., Taylor A.F., Maizels N. (2004). Intracellular transcription of G-rich DNAs induces formation of G-loops, novel structures containing G4 DNA. Genes Dev..

[35] Zhao J., Bacolla A., Wang G., Vasquez K.M. (2010). Non-B DNA structure-induced genetic instability and evolution. Cell. Mol. Life Sci..

[36] Raghavan S.C., Lieber M.R. (2004). Chromosomal translocations and non-B DNA structures in the human genome. Cell Cycle.

[37] Van Holde K., Zlatanova J. (1994). Unusual DNA structures, chromatin and transcription. Bioessays.

[38] Wang G., Vasquez K.M. (2009). Models for chromosomal replication-independent non-B DNA structure-induced genetic instability. Mol. Carcinog..

[39] Inagaki H., Ohye T., Kogo H., Kato T., Bolor H., Taniguchi M., Shaikh T.H., Emanuel B.S., Kurahashi H. (2009). Chromosomal instability mediated by non-B DNA: Cruciform conformation and not DNA sequence is responsible for recurrent translocation in humans. Genome Res..

[40] Schmidt M.H., Pearson C.E. (2016). Disease-associated repeat instability and mismatch repair. DNA Repair (Amst).

[41] Lahue R.S., Slater D.L. (2003). DNA repair and trinucleotide repeat instability. Front. Biosci..

[42] Bowater R.P., Wells R.D. (2001). The intrinsically unstable life of DNA triplet repeats associated with human hereditary disorders. Prog. Nucleic Acid Res. Mol. Biol..

[43] Wells R.D., Dere R., Hebert M.L., Napierala M., Son L.S. (2005). Advances in mechanisms of genetic instability related to hereditary neurological diseases. Nucleic Acids Res..

[44] Kearse M.G., Todd P.K. (2014). Repeat-associated non-AUG translation and its impact in neurodegenerative disease. Neurotherapeutics.

[45] Wiener F., Ohno S., Babonits M., Sumegi J., Wirschubsky Z., Klein G., Mushinski J.F., Potter M. (1984). Hemizygous interstitial deletion of chromosome 15 (band D) in three translocation-negative murine plasmacytomas. Proc. Natl. Acad. Sci. USA.

[46] Akasaka T., Akasaka H., Ueda C., Yonetani N., Maesako Y., Shimizu A., Yamabe H., Fukuhara S., Uchiyama T., Ohno H. (2000). Molecular and clinical features of non-Burkitt’s, diffuse large-cell lymphoma of B-cell type associated with the c-MYC/immunoglobulin heavy-chain fusion gene. J. Clin. Oncol..

[47] Kovalchuk A.L., Muller J.R., Janz S. (1997). Deletional remodeling of c-myc-deregulating chromosomal translocations. Oncogene.

[48] Joos S., Haluska F.G., Falk M.H., Henglein B., Hameister H., Croce C.M., Bornkamm G.W. (1992). Mapping chromosomal breakpoints of Burkitt’s t(8;14) translocations far upstream of c-myc. Cancer Res..

[49] Haluska F.G., Tsujimoto Y., Croce C.M. (1988). The t(8;14) breakpoint of the EW 36 undifferentiated lymphoma cell line lies 5′ of MYC in a region prone to involvement in endemic Burkitt’s lymphomas. Nucleic Acids Res..

[50] Saglio G., Grazia Borrello M., Guerrasio A., Sozzi G., Serra A., di Celle P.F., Foa R., Ferrarini M., Roncella S., Borgna Pignatti C. (1993). Preferential clustering of chromosomal breakpoints in Burkitt’s lymphomas and L3 type acute lymphoblastic leukemias with a t(8;14) translocation. Genes Chromosomes Cancer.

[51] Care A., Cianetti L., Giampaolo A., Sposi N.M., Zappavigna V., Mavilio F., Alimena G., Amadori S., Mandelli F., Peschle C. (1986). Translocation of c-myc into the immunoglobulin heavy-chain locus in human acute B-cell leukemia. A molecular analysis. EMBO J..

[52] Wilda M., Busch K., Klose I., Keller T., Woessmann W., Kreuder J., Harbott J., Borkhardt A. (2004). Level of MYC overexpression in pediatric Burkitt’s lymphoma is strongly dependent on genomic breakpoint location within the MYC locus. Genes Chromosomes Cancer.

[53] Rimokh R., Rouault J.P., Wahbi K., Gadoux M., Lafage M., Archimbaud E., Charrin C., Gentilhomme O., Germain D., Samarut J. (1991). A chromosome 12 coding region is juxtaposed to the MYC protooncogene locus in a t(8;12)(q24;q22) translocation in a case of B-cell chronic lymphocytic leukemia. Genes Chromosomes Cancer.

[54] Wolfl S., Wittig B., Rich A. (1995). Identification of transcriptionally induced Z-DNA segments in the human c-myc gene. Biochim. Biophys. Acta.

[55] Siddiqui-Jain A., Grand C.L., Bearss D.J., Hurley L.H. (2002). Direct evidence for a G-quadruplex in a promoter region and its targeting with a small molecule to repress c-MYC transcription. Proc. Natl. Acad. Sci. USA.

[56] Grand C.L., Han H., Munoz R.M., Weitman S., Von Hoff D.D., Hurley L.H., Bearss D.J. (2002). The cationic porphyrin TMPyP4 down-regulates c-MYC and human telomerase reverse transcriptase expression and inhibits tumor growth in vivo. Mol. Cancer Ther..

[57] Juranek S.A., Paeschke K. (2012). Cell cycle regulation of G-quadruplex DNA structures at telomeres. Curr. Pharm. Des..

[58] Capra J.A., Paeschke K., Singh M., Zakian V.A. (2010). G-quadruplex DNA sequences are evolutionarily conserved and associated with distinct genomic features in Saccharomyces cerevisiae. PLoS Comput. Biol..

[59] Kurahashi H., Inagaki H., Ohye T., Kogo H., Kato T., Emanuel B.S. (2006). Palindrome-mediated chromosomal translocations in humans. DNA Repair (Amst).

[60] Bacolla A., Tainer J.A., Vasquez K.M., Cooper D.N. (2016). Translocation and deletion breakpoints in cancer genomes are associated with potential non-B DNA-forming sequences. Nucleic Acids Res..

[61] Wang G., Vasquez K.M. (2004). Naturally occurring H-DNA-forming sequences are mutagenic in mammalian cells. Proc. Natl. Acad. Sci. USA.

[62] Wang G., Carbajal S., Vijg J., DiGiovanni J., Vasquez K.M. (2008). DNA structure-induced genomic instability in vivo. J. Natl. Cancer Inst..

[63] Wang G., Christensen L.A., Vasquez K.M. (2006). Z-DNA-forming sequences generate large-scale deletions in mammalian cells. Proc. Natl. Acad. Sci. USA.

[64] Wang G., Gaddis S., Vasquez K.M. (2013). Methods to detect replication-dependent and replication-independent DNA structure-induced genetic instability. Methods.

[65] Caskey C.T., Pizzuti A., Fu Y.H., Fenwick R.G., Nelson D.L. (1992). Triplet repeat mutations in human disease. Science.

[66] Martorell L., Monckton D.G., Gamez J., Johnson K.J., Gich I., Lopez de Munain A., Baiget M. (1998). Progression of somatic CTG repeat length heterogeneity in the blood cells of myotonic dystrophy patients. Hum. Mol. Genet..

[67] Wong L.J., Ashizawa T., Monckton D.G., Caskey C.T., Richards C.S. (1995). Somatic heterogeneity of the CTG repeat in myotonic dystrophy is age and size dependent. Am. J. Hum. Genet..

[68] Martorell L., Martinez J.M., Carey N., Johnson K., Baiget M. (1995). Comparison of CTG repeat length expansion and clinical progression of myotonic dystrophy over a five year period. J. Med. Genet..

[69] Wohrle D., Kennerknecht I., Wolf M., Enders H., Schwemmle S., Steinbach P. (1995). Heterogeneity of DM kinase repeat expansion in different fetal tissues and further expansion during cell proliferation in vitro: Evidence for a casual involvement of methyl-directed DNA mismatch repair in triplet repeat stability. Hum. Mol. Genet..

[70] Mirkin S.M., Smirnova E.V. (2002). Positioned to expand. Nat. Genet..

[71] Deng S.K., Yin Y., Petes T.D., Symington L.S. (2015). Mre11-Sae2 and RPA Collaborate to Prevent Palindromic Gene Amplification. Mol. Cell.

[72] Audry J., Maestroni L., Delagoutte E., Gauthier T., Nakamura T.M., Gachet Y., Saintomé C., Géli V., Coulon S. (2015). RPA prevents G-rich structure formation at lagging-strand telomeres to allow maintenance of chromosome ends. EMBO J..

[73] Deng S.K., Chen H., Symington L.S. (2015). Replication protein A prevents promiscuous annealing between short sequence homologies: Implications for genome integrity. Bioessays.

[74] Vlijm R., Mashaghi A., Bernard S., Modesti M., Dekker C. (2015). Experimental phase diagram of negatively supercoiled DNA measured by magnetic tweezers and fluorescence. Nanoscale.

[75] Nguyen B., Sokoloski J., Galletto R., Elson E.L., Wold M.S., Lohman T.M. (2014). Diffusion of human replication protein A along single-stranded DNA. J. Mol. Biol..

[76] Safa L., Delagoutte E., Petruseva I., Alberti P., Lavrik O., Riou J.F., Saintomé C. (2014). Binding polarity of RPA to telomeric sequences and influence of G-quadruplex stability. Biochimie.

[77] Zhang J., Zhou R., Inoue J., Mikawa T., Ha T. (2014). Single molecule analysis of Thermus thermophilus SSB protein dynamics on single-stranded DNA. Nucleic Acids Res..

[78] Kang S., Jaworski A., Ohshima K., Wells R.D. (1995). Expansion and deletion of CTG repeats from human disease genes are determined by the direction of replication in *E. coli*. Nat. Genet..

[79] Miret J.J., Pessoa-Brandao L., Lahue R.S. (1998). Orientation-dependent and sequence-specific expansions of CTG/CAG trinucleotide repeats in Saccharomyces cerevisiae. Proc. Natl. Acad. Sci. USA.

[80] Samadashwily G.M., Raca G., Mirkin S.M. (1997). Trinucleotide repeats affect DNA replication in vivo. Nat. Genet..

[81] Trinh T.Q., Sinden R.R. (1991). Preferential DNA secondary structure mutagenesis in the lagging strand of replication in *E. coli*. Nature.

[82] Hashem V.I., Sinden R.R. (2005). Duplications between direct repeats stabilized by DNA secondary structure occur preferentially in the leading strand during DNA replication. Mutat. Res..

[83] Iyer R.R., Wells R.D. (1999). Expansion and deletion of triplet repeat sequences in *Escherichia coli* occur on the leading strand of DNA replication. J. Biol. Chem..

[84] Pelletier R., Krasilnikova M.M., Samadashwily G.M., Lahue R., Mirkin S.M. (2003). Replication and expansion of trinucleotide repeats in yeast. Mol. Cell. Biol..

[85] Liu G., Chen X., Bissler J.J., Sinden R.R., Leffak M. (2010). Replication-dependent instability at (CTG) x (CAG) repeat hairpins in human cells. Nat. Chem. Biol..

[86] Maurer D.J., O’Callaghan B.L., Livingston D.M. (1996). Orientation dependence of trinucleotide CAG repeat instability in Saccharomyces cerevisiae. Mol. Cell. Biol..

[87] Freudenreich C.H., Stavenhagen J.B., Zakian V.A. (1997). Stability of a CTG/CAG trinucleotide repeat in yeast is dependent on its orientation in the genome. Mol. Cell. Biol..

[88] Hartenstine M.J., Goodman M.F., Petruska J. (2000). Base stacking and even/odd behavior of hairpin loops in DNA triplet repeat slippage and expansion with DNA polymerase. J. Biol. Chem..

[89] Cleary J.D., Nichol K., Wang Y.H., Pearson C.E. (2002). Evidence of cis-acting factors in replication-mediated trinucleotide repeat instability in primate cells. Nat. Genet..

[90] Shah K.A., Shishkin A.A., Voineagu I., Pavlov Y.I., Shcherbakova P.V., Mirkin S.M. (2012). Role of DNA polymerases in repeat-mediated genome instability. Cell Rep..

[91] Sarkar P.S., Chang H.C., Boudi F.B., Reddy S. (1998). CTG repeats show bimodal amplification in *E. coli*. Cell.

[92] Spiro C., Pelletier R., Rolfsmeier M.L., Dixon M.J., Lahue R.S., Gupta G., Park M.S., Chen X., Mariappan S.V., McMurray C.T. (1999). Inhibition of FEN-1 processing by DNA secondary structure at trinucleotide repeats. Mol. Cell.

[93] Iyer R.R., Pluciennik A., Rosche W.A., Sinden R.R., Wells R.D. (2000). DNA polymerase III proofreading mutants enhance the expansion and deletion of triplet repeat sequences in *Escherichia coli*. J. Biol. Chem..

[94] Kim N., Jinks-Robertson S. (2012). Transcription as a source of genome instability. Nat. Rev. Genet..

[95] Wittig B., Dorbic T., Rich A. (1991). Transcription is associated with Z-DNA formation in metabolically active permeabilized mammalian cell nuclei. Proc. Natl. Acad. Sci. USA.

[96] Wittig B., Wolfl S., Dorbic T., Vahrson W., Rich A. (1992). Transcription of human c-myc in permeabilized nuclei is associated with formation of Z-DNA in three discrete regions of the gene. EMBO J..

[97] Cerna A., Cuadrado A., Jouve N., Diaz de la Espina S.M., De la Torre C. (2004). Z-DNA, a new in situ marker for transcription. Eur. J. Histochem..

[98] Lombrana R., Almeida R., Alvarez A., Gomez M. (2015). R-loops and initiation of DNA replication in human cells: A missing link?. Front. Genet..

[99] Peleg M., Kopel V., Borowiec J.A., Manor H. (1995). Formation of DNA triple helices inhibits DNA unwinding by the SV40 large T-antigen helicase. Nucleic Acids Res..

[100] Kopel V., Pozner A., Baran N., Manor H. (1996). Unwinding of the third strand of a DNA triple helix, a novel activity of the SV40 large T-antigen helicase. Nucleic Acids Res..

[101] Hoyne P.R., Maher L.J. (2002). Functional studies of potential intrastrand triplex elements in the *Escherichia coli* genome. J. Mol. Biol..

[102] Hile S.E., Eckert K.A. (2004). Positive correlation between DNA polymerase alpha-primase pausing and mutagenesis within polypyrimidine/polypurine microsatellite sequences. J. Mol. Biol..

[103] Rao B.S. (1994). Pausing of simian virus 40 DNA replication fork movement in vivo by (dG-dA)n.(dT-dC)n tracts. Gene.

[104] Krasilnikova M.M., Mirkin S.M. (2004). Replication stalling at Friedreich’s ataxia (GAA)n repeats in vivo. Mol. Cell. Biol..

[105] Voineagu I., Narayanan V., Lobachev K.S., Mirkin S.M. (2008). Replication stalling at unstable inverted repeats: Interplay between DNA hairpins and fork stabilizing proteins. Proc. Natl. Acad. Sci. USA.

[106] Anand R.P., Shah K.A., Niu H., Sung P., Mirkin S.M., Freudenreich C.H. (2012). Overcoming natural replication barriers: Differential helicase requirements. Nucleic Acids Res..

[107] Voineagu I., Surka C.F., Shishkin A.A., Krasilnikova M.M., Mirkin S.M. (2009). Replisome stalling and stabilization at CGG repeats, which are responsible for chromosomal fragility. Nat. Struct. Mol. Biol..

[108] Wang Q., Liu J.Q., Chen Z., Zheng K.W., Chen C.Y., Hao Y.H., Tan Z. (2011). G-quadruplex formation at the 3′ end of telomere DNA inhibits its extension by telomerase, polymerase and unwinding by helicase. Nucleic Acids Res..

[109] Han H., Hurley L.H., Salazar M. (1999). A DNA polymerase stop assay for G-quadruplex-interactive compounds. Nucleic Acids Res..

[110] Paeschke K., Capra J.A., Zakian V.A. (2011). DNA replication through G-quadruplex motifs is promoted by the Saccharomyces cerevisiae Pif1 DNA helicase. Cell.

[111] Wang G., Zhao J., Vasquez K.M. (2009). Methods to determine DNA structural alterations and genetic instability. Methods.

[112] Tornaletti S., Reines D., Hanawalt P.C. (1999). Structural characterization of RNA polymerase II complexes arrested by a cyclobutane pyrimidine dimer in the transcribed strand of template DNA. J. Biol. Chem..

[113] Ditlevson J.V., Tornaletti S., Belotserkovskii B.P., Teijeiro V., Wang G., Vasquez K.M., Hanawalt P.C. (2008). Inhibitory effect of a short Z-DNA forming sequence on transcription elongation by T7 RNA polymerase. Nucleic Acids Res..

[114] Belotserkovskii B.P., De Silva E., Tornaletti S., Wang G., Vasquez K.M., Hanawalt P.C. (2007). A triplex-forming sequence from the human c-MYC promoter interferes with DNA transcription. J. Biol. Chem..

[115] Pandey S., Ogloblina A.M., Belotserkovskii B.P., Dolinnaya N.G., Yakubovskaya M.G., Mirkin S.M., Hanawalt P.C. (2015). Transcription blockage by stable H-DNA analogs in vitro. Nucleic Acids Res..

[116] Belotserkovskii B.P., Liu R., Tornaletti S., Krasilnikova M.M., Mirkin S.M., Hanawalt P.C. (2010). Mechanisms and implications of transcription blockage by guanine-rich DNA sequences. Proc. Natl. Acad. Sci. USA.

[117] Mirkin E.V., Mirkin S.M. (2005). Mechanisms of transcription-replication collisions in bacteria. Mol. Cell. Biol..

[118] Olavarrieta L., Martinez-Robles M.L., Hernandez P., Krimer D.B., Schvartzman J.B. (2002). Knotting dynamics during DNA replication. Mol. Microbiol..

[119] Kim N., Jinks-Robertson S. (2009). dUTP incorporation into genomic DNA is linked to transcription in yeast. Nature.

[120] Thys R.G., Lehman C.E., Pierce L.C., Wang Y.H. (2015). DNA secondary structure at chromosomal fragile sites in human disease. Curr. Genom..

[121] Durkin S.G., Glover T.W. (2007). Chromosome fragile sites. Annu. Rev. Genet..

[122] Debacker K., Kooy R.F. (2007). Fragile sites and human disease. Hum. Mol. Genet..

[123] Arlt M.F., Durkin S.G., Ragland R.L., Glover T.W. (2006). Common fragile sites as targets for chromosome rearrangements. DNA Repair (Amst).

[124] Sutherland G.R. (2003). Rare fragile sites. Cytogenet. Genome Res..

[125] Glover T.W. (1998). Instability at chromosomal fragile sites. Recent Results Cancer Res..

[126] Ohta M., Inoue H., Cotticelli M.G., Kastury K., Baffa R., Palazzo J., Siprashvili Z., Mori M., McCue P., Druck T. (1996). The FHIT gene, spanning the chromosome 3p14.2 fragile site and renal carcinoma-associated t(3;8) breakpoint, is abnormal in digestive tract cancers. Cell.

[127] Calin G.A., Sevignani C., Dumitru C.D., Hyslop T., Noch E., Yendamuri S., Shimizu M., Rattan S., Bullrich F., Negrini M. (2004). Human microRNA genes are frequently located at fragile sites and genomic regions involved in cancers. Proc. Natl. Acad. Sci. USA.

[128] Smith D.I., Zhu Y., McAvoy S., Kuhn R. (2006). Common fragile sites, extremely large genes, neural development and cancer. Cancer Lett..

[129] Helmrich A., Ballarino M., Tora L. (2011). Collisions between replication and transcription complexes cause common fragile site instability at the longest human genes. Mol. Cell.

[130] Le Tallec B., Millot G.A., Blin M.E., Brison O., Dutrillaux B., Debatisse M. (2013). Common fragile site profiling in epithelial and erythroid cells reveals that most recurrent cancer deletions lie in fragile sites hosting large genes. Cell Rep..

[131] Barlow J.H., Faryabi R.B., Callen E., Wong N., Malhowski A., Chen H.T., Gutierrez-Cruz G., Sun H.W., McKinnon P., Wright G. (2013). Identification of early replicating fragile sites that contribute to genome instability. Cell.

[132] Brambati A., Colosio A., Zardoni L., Galanti L., Liberi G. (2015). Replication and transcription on a collision course: Eukaryotic regulation mechanisms and implications for DNA stability. Front Genet.

[133] Sankar T.S., Wastuwidyaningtyas B.D., Dong Y., Lewis S.A., Wang J.D. (2016). The nature of mutations induced by replication-transcription collisions. Nature.

[134] Herold S., Herkert B., Eilers M. (2009). Facilitating replication under stress: An oncogenic function of MYC?. Nat. Rev. Cancer.

[135] Shigemi Z., Baba Y., Hara N., Matsuhiro J., Kagawa H., Watanabe T., Fujimuro M. (2016). Effects of ER stress on unfolded protein responses, cell survival, and viral replication in primary effusion lymphoma. Biochem. Biophys. Res. Commun..

[136] Barone G., Staples C.J., Ganesh A., Patterson K.W., Bryne D.P., Myers K.N., Patil AA., Eyers C.E., Maslen S., Skehel J.M. (2016). Human CDK18 promotes replication stress signaling and genome stability. Nucleic Acids Res..

[137] Palou G., Palou R., Zeng F., Vashisht A.A., Wohlschlegel J.A., Quintana D.G. (2015). Three Different Pathways Prevent Chromosome Segregation in the Presence of DNA Damage or Replication Stress in Budding Yeast. PLoS Genet..

[138] Chiker S., Pennaneach V., Loew D., Dingli F., Biard D., Cordelieres F.P., Gemble S., Vacher S., Bieche I., Hall J. (2015). Cdk5 promotes DNA replication stress checkpoint activation through RPA-32 phosphorylation, and impacts on metastasis free survival in breast cancer patients. Cell Cycle.

[139] Srinivasan S.V., Dominguez-Sola D., Wang L.C., Hyrien O., Gautier J. (2013). Cdc45 is a critical effector of myc-dependent DNA replication stress. Cell Rep.

[140] Neelsen K.J., Zanini I.M., Herrador R., Lopes M. (2013). Oncogenes induce genotoxic stress by mitotic processing of unusual replication intermediates. J. Cell Biol..

[141] Jones R.M., Mortusewicz O., Afzal I., Lorvellec M., Garcia P., Helleday T., Petermann E. (2013). Increased replication initiation and conflicts with transcription underlie Cyclin E-induced replication stress. Oncogene.

[142] Yu D.S., Zhao R., Hsu E.L., Cayer J., Ye F., Guo Y., Shyr Y., Cortez D. (2010). Cyclin-dependent kinase 9-cyclin K functions in the replication stress response. EMBO Rep..

[143] Maya-Mendoza A., Ostrakova J., Kosar M., Hall A., Duskova P., Mistrik M., Merchut-Maya J.M., Hodny Z., Bartkova J., Christensen C. (2015). Myc and Ras oncogenes engage different energy metabolism programs and evoke distinct patterns of oxidative and DNA replication stress. Mol. Oncol..

[144] Tsantoulis P.K., Kotsinas A., Sfikakis P.P., Evangelou K., Sideridou M., Levy B., Mo L., Kittas C., Wu X.R., Papavassiliou A.G. (2008). Oncogene-induced replication stress preferentially targets common fragile sites in preneoplastic lesions. A genome-wide study. Oncogene.

[145] Gaillard H., Garcia-Muse T., Aguilera A. (2015). Replication stress and cancer. Nat. Rev. Cancer.

[146] Zeman M.K., Cimprich K.A. (2014). Causes and consequences of replication stress. Nat. Cell Biol..

[147] Bertoli C., Klier S., McGowan C., Wittenberg C., de Bruin R.A. (2013). Chk1 inhibits E2F6 repressor function in response to replication stress to maintain cell-cycle transcription. Curr. Biol..

[148] Bertoli C., Herlihy A.E., Pennycook B.R., Kriston-Vizi J., de Bruin R.A. (2016). Sustained E2F-Dependent Transcription Is a Key Mechanism to Prevent Replication-Stress-Induced DNA Damage. Cell Rep..

[149] Kotsantis P., Silva L.M., Irmscher S., Jones R.M., Folkes L., Gromak N., Petermann E. (2016). Increased global transcription activity as a mechanism of replication stress in cancer. Nat. Commun..

[150] Stork C.T., Bocek M., Crossley M.P., Sollier J., Sanz L.A., Chedin F., Swigut T., Cimprich K.A. (2016). Co-transcriptional R-loops are the main cause of estrogen-induced DNA damage. Elife.

[151] Macheret M., Halazonetis T.D. (2015). DNA replication stress as a hallmark of cancer. Annu. Rev. Pathol..

[152] Gomez D., O’Donohue M.F., Wenner T., Douarre C., Macadre J., Koebel P., Giraud-Panis M.J., Kaplan H., Kolkes A., Shin-ya K. (2006). The G-quadruplex ligand telomestatin inhibits POT1 binding to telomeric sequences in vitro and induces GFP-POT1 dissociation from telomeres in human cells. Cancer Res..

[153] Tauchi T., Shin-Ya K., Sashida G., Sumi M., Nakajima A., Shimamoto T., Ohyashiki J.H., Ohyashiki K. (2003). Activity of a novel G-quadruplex-interactive telomerase inhibitor, telomestatin (SOT-095), against human leukemia cells: Involvement of ATM-dependent DNA damage response pathways. Oncogene.

[154] Tauchi T., Shin-ya K., Sashida G., Sumi M., Okabe S., Ohyashiki J.H., Ohyashiki K. (2006). Telomerase inhibition with a novel G-quadruplex-interactive agent, telomestatin: In vitro and in vivo studies in acute leukemia. Oncogene.

[155] Temime-Smaali N., Guittat L., Sidibe A., Shin-ya K., Trentesaux C., Riou J.F. (2009). The G-quadruplex ligand telomestatin impairs binding of topoisomerase IIIalpha to G-quadruplex-forming oligonucleotides and uncaps telomeres in ALT cells. PLoS ONE.

[156] Hasegawa D., Okabe S., Okamoto K., Nakano I., Shin-ya K., Seimiya H. (2016). G-quadruplex ligand-induced DNA damage response coupled with telomere dysfunction and replication stress in glioma stem cells. Biochem. Biophys. Res. Commun..

[157] Taylor E.M., Lindsay H.D. (2016). DNA replication stress and cancer: Cause or cure?. Future Oncol..

[158] Puigvert J.C., Sanjiv K., Helleday T. (2016). Targeting DNA repair, DNA metabolism and replication stress as anti-cancer strategies. FEBS J..

[159] Berti M., Vindigni A. (2016). Replication stress: Getting back on track. Nat. Struct. Mol. Biol..

[160] Kotsantis P., Jones R.M., Higgs M.R., Petermann E. (2015). Cancer therapy and replication stress: Forks on the road to perdition. Adv. Clin. Chem..

[161] Rizzo A., Salvati E., Porru M., D’Angelo C., Stevens M.F., D’Incalci M., Leonetti C., Gilson E., Zupi G., Biroccio A. (2009). Stabilization of quadruplex DNA perturbs telomere replication leading to the activation of an ATR-dependent ATM signaling pathway. Nucleic Acids Res..

